# Supplementary material for: An open-source framework for large-scale, flexible evaluation of biomedical text mining systems
Source: J Biomed Discov Collab. 2008 Jan 29;3:1. doi: 10.1186/1747-5333-3-1 (PMC2276192; doi:10.1186/1747-5333-3-1)
Supplement: Additional file 1 — Evaluation platform source code. The additional file contains the software evaluation framework discussed in this paper. The most current release can be downloaded from SourceForge.net [1]. See the accompanying README file for instructions on installation and use. Also included in the distribution is a collection of UIMA wrappers for some commonly used BioNLP tools and annotated corpora. [file 1747-5333-3-1-S1.zip › bionlp_uima_repository-1.0.1/docs/api/src-html/edu/uchsc/ccp/util/nlp/mention/ComplexSlotMentionTypes.html]

```
001    package edu.uchsc.ccp.util.nlp.mention;
002    
003    public interface ComplexSlotMentionTypes {
004    
005        /** ComplexSlotMention Identifier - if this changes, Annotation DB must be adjusted accordingly */
006        public static final String COMPLEX_SLOT_MENTION_TYPE = "complex slot mention";
007        
008    //    public static final String TOKEN_TYPED_DEPENDENCIES = "typedDependencies"; 
009        
010        public static final String SUBSTITUTION_MUTANT_ELEMENT = "mutant element";
011        public static final String SUBSTITUTION_WILDTYPE_ELEMENT = "wild type element";
012        public static final String DELETION_DELETED_ELEMENT = "deleted element";
013        public static final String INSERTION_INSERTED_ELEMENT = "inserted element";
014        public static final String INSERTION_INSERTION_START = "insertion start";
015        public static final String BIOLOGICAL_SEQUENCE_ELEMENT_POSITION_IN_SEQUENCE = "position in sequence";
016        public static final String BIOLOGICAL_SUBSEQUENCE_START_ELEMENT = "start element";
017        public static final String BIOLOGICAL_SUBSEQUENCE_END_ELEMENT = "end element";
018    }
```
